# Supplementary material for: The impact of US Government Stop Work Order on HIV epidemic trajectory in Zimbabwe
Source: PLOS Glob Public Health. 2026 May 6;6(5):e0006288. doi: 10.1371/journal.pgph.0006288 (PMC13148674; doi:10.1371/journal.pgph.0006288)
Supplement: Attachment — Submitted filename: PLOS Review.docx [file pgph.0006288.s003.docx]

The paper characterizes Zimbabwe’s reliance on USG funding using 2020 data. While this may be from the most recently published National AIDS Spending Assessment, more recent figures are certainly available. For instance, the [US embassy](https://zw.usembassy.gov/pepfars-20-years-of-impact-in-zimbabwe/) reports that “In 2023 alone, PEPFAR has invested more than $200 million to fund and deliver HIV/AIDS programs in Zimbabwe and save Zimbabwean lives.”

The paper uses past tense to describe the USG reliance: “Zimbabwe was highly dependent on United States Government (USG) funding…”, although this is still true today, despite the funding freeze and subsequent cuts. Suggest to use present tense.

In general, it would be good to characterize the nature of the PEPFAR cuts in more detail. It would seem that about $2.9 billion out of the $6 billion authorized has been released for 2025, with a similar amount proposed for FY26 spending, as per [NY Times](https://www.nytimes.com/2025/08/21/health/hiv-aids-pepfar-funding-trump.html).

Can the financial implications of the Stop Work Order in Zimbabwe and subsequent cuts be estimated, given that there are [publicly available datasets](https://www.cgdev.org/blog/new-estimates-usaid-cuts) of the cancelled USAID awards? [Some report](https://cite.org.zw/67m-gap-threatens-zimbabwes-hiv-prevention-efforts/) that “Zimbabwe will need at least US$67 million in 2026 to maintain its HIV response following a partial suspension of U.S. PEPFAR support.” The MOHCC may have more accurate estimates.

There may be a need to modify the modelled scenarios. Instead of, or in addition to, modelling the effect of stopping PEPFAR funding for prevention, and stopping PEPFAR funding for treatment, could there be a scenario based on stopping funding for the interventions specified in [the waiver](https://www.unaids.org/sites/default/files/media_asset/GHSD_PEPFAR-Limited-Waiver-Approved-Activities.pdf)? Further, since the cuts to Zimbabwe’s current Global Fund allocation [are known](https://women4gf.org/2025/07/08/%F0%9F%93%89-budget-cuts-in-gc7/) for GC7 (-11%) should this perhaps be modelled as well, or instead of, the worst-case 30% cut?

Do all the scenarios assume no mitigation from government or other donors to close funding gaps? If this is the case it should be clearly stated in the methods and in the conclusion. In fact, the entire paper is silent on any policy recommendations for the government or other partners.

“…by reaching 95-99-96 in 2023” can be updated with 2024, which is available data now.

Suggest replacing “Mother to Child transmission” with “vertical transmission”, in line with recent [language guidelines](https://www.unaids.org/sites/default/files/media_asset/2024-terminology-guidelines_en.pdf).

“The Presidents Emergency Plan for AIDS Relief (PEPFAR)” should be “The **U.S.** Presidents Emergency Plan for AIDS Relief (PEPFAR)”

“The stop order on USAID funding was expected to have a significant negative impact on the Zimbabwean healthcare system” seems odd to be in past tense again. Suggest “is expected to have…”

“Zimbabwe has lost at least 27% of total annual external funding for health”…from when to when? Is this in 2025 alone?

“There are 1.3 million people living with HIV, including 15,000 new infections annually so any Stop Work Orders have the potential to severely destabilize the HIV response in the country.” The size of the epidemic doesn’t necessarily mean that a SOW will be significantly felt. Suggest reworking this to show the reliance on USG for key interventions. For instance, of the 1.3m living with HIV, PEPFAR provided viral load testing for 867,413 of them in 2023.

“Although a waiver was issued, 10 February, allowing the continuation” is awkward phrasing. Suggest “Although a waiver was issued on 10 February 2025, allowing the continuation…”

The background section says nothing about the government response to the SOW and subsequent cuts.

“PEPAFR” is misspelled on page 6.

At times, Global Fund is abbreviated to “GF” which is not common or recommended. Suggest using the full name upon first use, and abbreviating to Global Fund thereafter: “Global Fund to Fight AIDS, Tuberculosis and Malaria (Global Fund)”.

“AIDS related deaths” should be hyphenated: “AIDS-related deaths”

“The increase in new HIV infections is against the global target of reducing new HIV infections by 90% from 2010 baseline(Galvani et al. 2018)(Kumar et al. 2022)(Lyden et al. 2023), however given the current economic and funding landscape there maybe need to revise the targets” – the references in the middle of the sentence are very odd and not linked to the content. Suggest they are deleted.

Instead of the policy recommendation to revise prevention targets, which UNAIDS [does not intend to do](https://www.unaids.org/sites/default/files/2025-05/20250328_recommended_2030_HIV_targets_livedocument_en_13_May_2025.pdf), it may be more relevant to discuss the revised resource needs estimates, which are significant. See the most recent [Global AIDS Update](https://www.unaids.org/sites/default/files/media_asset/2024-unaids-global-aids-update_en.pdf), which says “Preliminary estimates from UNAIDS indicate that achieving the global targets will require annual resources in 2030 of US$ 21.9 billion in low- and middle-income countries, down from the previously estimated US$ 29.3 billion”. Mostly due to efficiencies in ART unit costs, other commodities, and service delivery modalities, this makes the 2030 targets in a constrained environment somewhat more feasible.

There are a lot of typographical errors, especially in the discussion, where spaces are missing between text and in-text citations. The language in the discussion also needs significant revising for clarity, academic tone, and logical sense.

“cuts on targets will not follow a leaner approach” – do the authors mean “linear” here?

“The country has a target of reducing AIDS related deaths by 50% from a baseline of 60,000 in 2018(NAC 2021), the Trump orders will defeat the cause by increasing AIDS related deaths as a result of reduced treatment coverage”. This should be two separate sentences. Language like “defeat the cause” is not appropriate for a journal publication.

PLOS Global Public Health uses Vancouver style referencing, which this manuscript has not adhered to. The revised version should follow the journal’s format.

“The study AIDS related deaths scenario estimates increase were higher (300%) than the ones from South African modeling study performed for by Ghandi et al which estimated 110% increase in mortality(Gandhi 2025) likely because of treatment coverage that will be greatly reduced”. This is very unclear. Perhaps what it means to say is that PEPFAR cuts are modelled to have a greater effect on AIDS-related deaths in Zimbabwe as compared to South Africa (Gandhi, 2025) due to Zimbabwe’s stronger reliance on external partners to fund its treatment program.

The discussion must consider the potential for domestic or other funding to fill gaps, review what government has done (or not done) in response, and suggest sustainability options. For instance, Zimbabwe is developing an HIV Response Sustainability Roadmap which should be mentioned.

The discussion should content with [recent NY Times reporting](https://www.nytimes.com/2025/07/23/health/pepfar-shutdown.html) that Zimbabwe may be on a three-to-four-year timeline to completely wind down all PEPFAR support. This makes the complete funding cessation scenario a frightening but potentially realistic one.

There are a few key references that are not discussed in the paper and should be:

- Jimu, C. (2025). The precarious future of HIV and AIDS programs in Zimbabwe following international funding freeze: Impact and strategic interventions. *Journal of Epidemiology and Global Health*, *15*(1), 79.
- Musuka, G., Makoni, T., & Dzinamarira, T. (2025). Towards a sustainable HIV response: strengthening Zimbabwe's domestic financing for HIV programs amid declining donor support. *Frontiers in Health Services*, *5*, 1558992.
- Ten Brink, D., Martin-Hughes, R., Bowring, A. L., Wulan, N., Burke, K., Tidhar, T., ... & Scott, N. (2025). Impact of an international HIV funding crisis on HIV infections and mortality in low-income and middle-income countries: a modelling study. *The Lancet HIV*, *12*(5), e346-e354.
