# Supplementary material for: The impact of US Government Stop Work Order on HIV epidemic trajectory in Zimbabwe
Source: PLOS Glob Public Health. 2026 May 6;6(5):e0006288. doi: 10.1371/journal.pgph.0006288 (PMC13148674; doi:10.1371/journal.pgph.0006288)
Supplement: Attachment — Submitted filename: Responses to Reviewers_Second round of comments.docx [file pgph.0006288.s005.docx]

**Comments**
1. In the abstract "waste case scenario" should be stated as "worst case scenario"

**Response:** Corrected, although the correction was done in the first round of the comments.

2. Is Stop Work Orders referred to as plural or singular? Some places are plural, others singular. Use consistent grammatical number/syntax/morphology.

**Response:** Corrected, and we used singular in all instances.

3. Has the Goals model ever been validated, i.e., how much confidence do we have that the projections resulting from the Goals tool are sufficiently close the true parameters of HIV burden?

**Response:** The Goals model has been validated and published. Validation of the application of the model to Zimbabwe is done by comparing the Goals model output with data on prevalence by population group over time.

The Goals application to Zimbabwe was validated by comparing the model estimates of prevalence over time by population group with available prevalence data from national surveys in 2005-06 (DHS), 2010-11 (DHS), 2015-16 (DHS), 2015-16 (PHIA) and 2020 (PHIA).

4. Does the model generate uncertainty intervals/confidence intervals of the projected estimates of HIV burden? If so, it will be informative for readers to see the range of uncertainty.

**Response:** We used the five scenarios to represent the uncertainty of the projections since the range of future outcomes is much more sensitive to assumptions about future funding than to the statistical variation around projections.

5. In Figure 4, the legend does not show what scenario is represented by the blue line.

**Response:** Corrected in the first round of comments. Note that the business-as-usual scenario in Figure 3 and 4 is overshadowed by the Only HIV prevention removed scenario. There is no difference in outputs for the two scenarios.

6. The discussion appears a bit too lean. Given the magnitude of the looming burden of HIV resulting from the cut in funding for HIV programming, the research team should discuss what the government of Zimbabwe should proactively do. Instead of the government of Zimbabwe waiting (as sitting ducks) for funding to somehow miraculously be reinstated, the research team should offer suggestions. For example, how can the AIDS levy or a significant proportion of the government budget to be redirected to fill the gap in funding? Suggestions for the discussion are: <https://www.sciencedirect.com/science/article/abs/pii/S1055329016300401>;

**Response:** Discussions on government of Zimbabwe is doing and what it should do was added. Suggested discussions were also added, although some were added in the previous round of comments.

7. Another suggestion for the discussion is how to engage with the population so that they also grasp the gravity of the funding cut for HIV programming.

**Response:** We have included in the discussions that the government is engaging with policymakers and programmers on how to close the funding gap. The ongoing discussions on ways to mobilise resources were also included.
